# Supplementary material for: Unique Gut Microbiome Signatures among Adult Patients with Moderate to Severe Atopic Dermatitis in Southern Chinese
Source: Int J Mol Sci. 2023 Aug 16;24(16):12856. doi: 10.3390/ijms241612856 (PMC10454836; doi:10.3390/ijms241612856)
Supplement: Supplementary file 1 [file ijms-24-12856-s001.zip › Supplementary_Figures_Legends.pdf]

Supplementary Figures

Figure legends:

Figure S1 Flowchart of the experimental design.

Figure S2 Beta diversity analysis between the AD and normal groups using data denoised by mbDenoise. Data ordination through mbDenoise-zinb model based and (A) without algorithm-based and with algorithm-based (B)PCA, (C)PCoA and (D) t-SNE. Beta diversity was assessed using permutational multivariate analysis of variance (PERMANOVA).

Figure S3 Beta diversity analysis between the AD and normal groups using data normalized by mbImpute. Input of PCA were log-transformed.

Figure S4 Boxplot of B/F ration across groups, *p* value was calculated using Kruskal-Wallis test.

Figure S5 Bar plot of LEfse analysis results in the gut microbiome of Mild_AD, Severe_AD, and normal groups.

Figure S6 Receiver-operating characteristic (ROC) plots for the gut microbial signature selected by LEfSe.
