# Supplementary material for: Unique Gut Microbiome Signatures among Adult Patients with Moderate to Severe Atopic Dermatitis in Southern Chinese
Source: Int J Mol Sci. 2023 Aug 16;24(16):12856. doi: 10.3390/ijms241612856 (PMC10454836; doi:10.3390/ijms241612856)
Supplement: Supplementary file 1 [file ijms-24-12856-s001.zip › Supplementary_Tables.pdf]

Supplementary Tables

Table S1 Characteristics in Mild_AD group, Severe_AD group, and normal group.

Parameters		Mild_AD (n = 53)		Severe_AD(n = 51)		Normal (n = 130)		p value[1]
		$mean \pm sd$	n	$mean \pm sd$	n	$mean \pm sd$	n	
Age		44.8 ± 14.6		43.9 ± 15.7		47.7 ± 15.1		0.413
Sex[2]	Female		20		30		41	0.403
	Male		16		13		27	
Allergy[2]	Yes		11		17		10	**0.002**
	No		18		15		50	
	Not sure		7		11		8	
Food allergy[2]	Yes		4		8		5	0.183
	No		21		35		63	
Non-food allergy[2]	Yes		6		11		5	0.120
	No		30		32		63	
Diarrhea[2]	Yes		8		9		18	0.774
	No		28		34		50	
Constipation[2]	Yes		10		16		30	0.261
	No		26		27		38	
BMI[2]		22.7 ± 6.6		22.6 ± 4.0		23.1 ± 3.8		0.815
Overweight (BMI > 25)[2]	Yes		12		8		19	0.523
	No		21		33		44	
	Not sure		3		2		5	

[1] p value was calculated using Chi-square test and Wilcoxon rank-sum test.

[2] 147 of 234 participants provided the clinical information of allergy history, prevalence of diarrhea and constipation, BMI (both height and weight) for subsequent patient characteristics analysis.

Table S2 Summary of alpha diversity analysis results of different grouping schemes

Measure	2 Groups		3 Groups					
	Normal VS AD		Normal VS Mild AD		Normal VS Severe AD		Mild AD VS Severe AD	
	p value	Sig.	*p* value	Sig.	*p* value	Sig.	*p* value	Sig.
Observed OTUs	0.928		0.789		0.874		0.817	
Chao 1	0.859		0.701		0.878		0.777	
ACE	0.766		0.551		0.834		0.673	
Shannon diversity index	0.285		0.249		0.743		0.734	
InvSimpson index	0.369		0.225		0.830		0.483	
Fisher	0.901		0.799		0.933		0.876	
Faith pd	0.844		0.831		0.792		0.842	

Abbreviation: Sig., Significant

Sig. codes: * $p < 0.05$; ** $p < 0.01$; *** $p < 0.001$

Table S3 Beta diversity analysis results based on four different metrics across groups

Group	Metric	*p* value	Sig.
Normal VS AD	Jaccard distance metric	0.001	***
	Bray-Curtis distance metric	0.001	***
	Unweighted UniFrac distance metric	0.025	*

	Weighted UniFrac distance metric	0.181	
Normal VS Mild AD	Jaccard distance metric	0.025	*
	Bray-Curtis distance metric	0.030	*
	Unweighted UniFrac distance metric	0.343	
	Weighted UniFrac distance metric	0.082	
Normal VS Severe AD	Jaccard distance metric	0.001	***
	Bray-Curtis distance metric	0.001	***
	Unweighted UniFrac distance metric	0.031	*
	Weighted UniFrac distance metric	0.429	
Mild AD VS Severe AD	Jaccard distance metric	0.119	
	Bray-Curtis distance metric	0.103	
	Unweighted UniFrac distance metric	0.769	
	Weighted UniFrac distance metric	0.139	

Abbreviation: Sig., Significant

Sig. codes: * $p < 0.05$; ** $p < 0.01$; *** $p < 0.001$

Table S4 Relative abundance of species at the phylum level in AD and normal groups

Kingdom	Phylum	AD (mean)	Normal (mean)	W value
Bacteria	Firmicutes	0.5738	0.5816	1
	Bacteroidota	0.3243	0.3207	0
	Actinobacteriota	0.0618	0.0546	0

	Proteobacteria	0.0275	0.0244	0
	Fusobacteria	0.0029	0.0074	0
	Desulfobacterota	0.0046	0.0045	0
	Verrucomicrobiota	0.0043	0.0041	0
	Cyanobacteria	0.0002	0.0020	0
	Elusimicrobiota	0	3.94e-06	0
	Synergistota	0.0003	0.0003	0
	Unclassified	5.67e-05	2.56e-05	0
	Campilobacterota	2.96e-05	1.97e-05	0
Archaea	Euryarchaeota	0.0003	0.0004	0
	Halobacterota	0	7.89e-06	0
	Thermoplasmatota	3.45e-05	0	0

W value was calculated using ANCOM

Table S5 Gut microbial biomarkers selected by LEfSe analysis

Bacteria	Group	LDA score	*p* value
g_Romboutsia	Normal	3.49	0.002
g_Clostridium_sensu_stricto_1	Normal	3.16	0.0008
f_Butyricicoccaceae	Normal	2.10	0.008
f_Erysipelotrichaceae	Normal	3.54	0.006
Blautia	AD	3.78	0.009

Butyricicoccus	AD	3.28	0.003
Lachnoclostridium	AD	3.22	0.045
Eubacterium_hallii_group	AD	3.12	0.015
Erysipelatoclostridium	AD	3.03	0.019
Megasphaera	AD	2.94	0.017
Oscillibacter	AD	2.87	0.019
Flavonifractor	AD	2.62	0.026
f_Oscillospiraceae	AD	2.93	0.001

Table S6 Differentially enriched predicted functional pathways across groups using PICRUSt2 and LEfSe analysis.

MetaCyc ID	MetaCyc pathway	Group	LDA score	*p* value
PWY-4984	Urea cycle	Normal	2.00	0.001
PWY490-3	Nitrate reduction VI (assimilatory)	Normal	2.08	0.045
PWY-5659	GDP-mannose biosynthesis	Normal	2.13	0.01
GLYCOLYSIS	Glycolysis I (from glucose 6-phosphate)	Normal	2.14	0.038

PWY-2941	L-lysine biosynthesis II	Normal	2.17	0.01
PWY-6630	Superpathway of L-tyrosine biosynthesis	Normal	2.28	0.004
PWY-6628	Superpathway of L-phenylalanine biosynthesis	Normal	2.29	0.004
PWY-7315	dTDP-N-acetylthomosamine biosynthesis	AD	2.01	0.017
PWY-7242	D-fructuronate degradation	AD	2.09	0.027
P164-PWY	Purine nucleobases degradation I (anaerobic)	AD	2.10	0.005
PWY-1861	Formaldehyde assimilation II (RuMP Cycle)	AD	2.11	0.009
GLYCOLYSIS-E-D	Superpathway of glycolysis and Entner-Doudoroff	AD	2.12	0.035
P441-PWY	Superpathway of N-acetylneuraminate degradation	AD	2.12	0.044
METH-ACETATE-PWY	Methanogenesis from acetate	AD	2.16	0.001
PWY-6608	Guanosine nucleotides degradation III	AD	2.24	0.004
PWY-6353	Purine nucleotides degradation II (aerobic)	AD	2.32	0.0003
SALVADEHYPOX-PWY	Adenosine nucleotides degradation II	AD	2.37	0.0003
PWY-5971	Palmitate biosynthesis II (bacteria and plants)	AD	2.38	0.027
PRYIDOXSYN-PWY	Pyridoxal 5'-phosphate biosynthesis I	Mild_AD	2.62	0.006
PWY0-845	Superpathway of pyridoxal 5'-phosphate biosynthesis and salvage	Mild_AD	2.67	0.006
PWY-6353	Purine nucleotides degradation II (aerobic)	Severe_AD	2.53	0.0002
SALVADEHYPOX-PWY	Adenosine nucleotides degradation II	Severe_AD	2.57	0.0002
