# Supplementary figures and images for: Unique Gut Microbiome Signatures among Adult Patients with Moderate to Severe Atopic Dermatitis in Southern Chinese
Source: Int J Mol Sci. 2023 Aug 16;24(16):12856. doi: 10.3390/ijms241612856 (PMC10454836; doi:10.3390/ijms241612856)

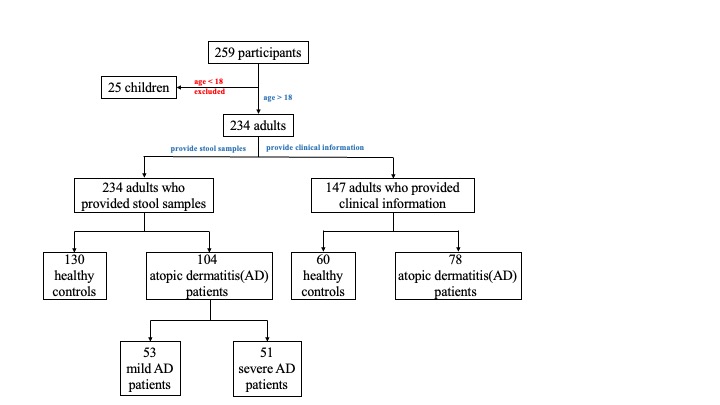

Supplement: Supplementary file 1 [file ijms-24-12856-s001.zip › FigureS1.jpg]

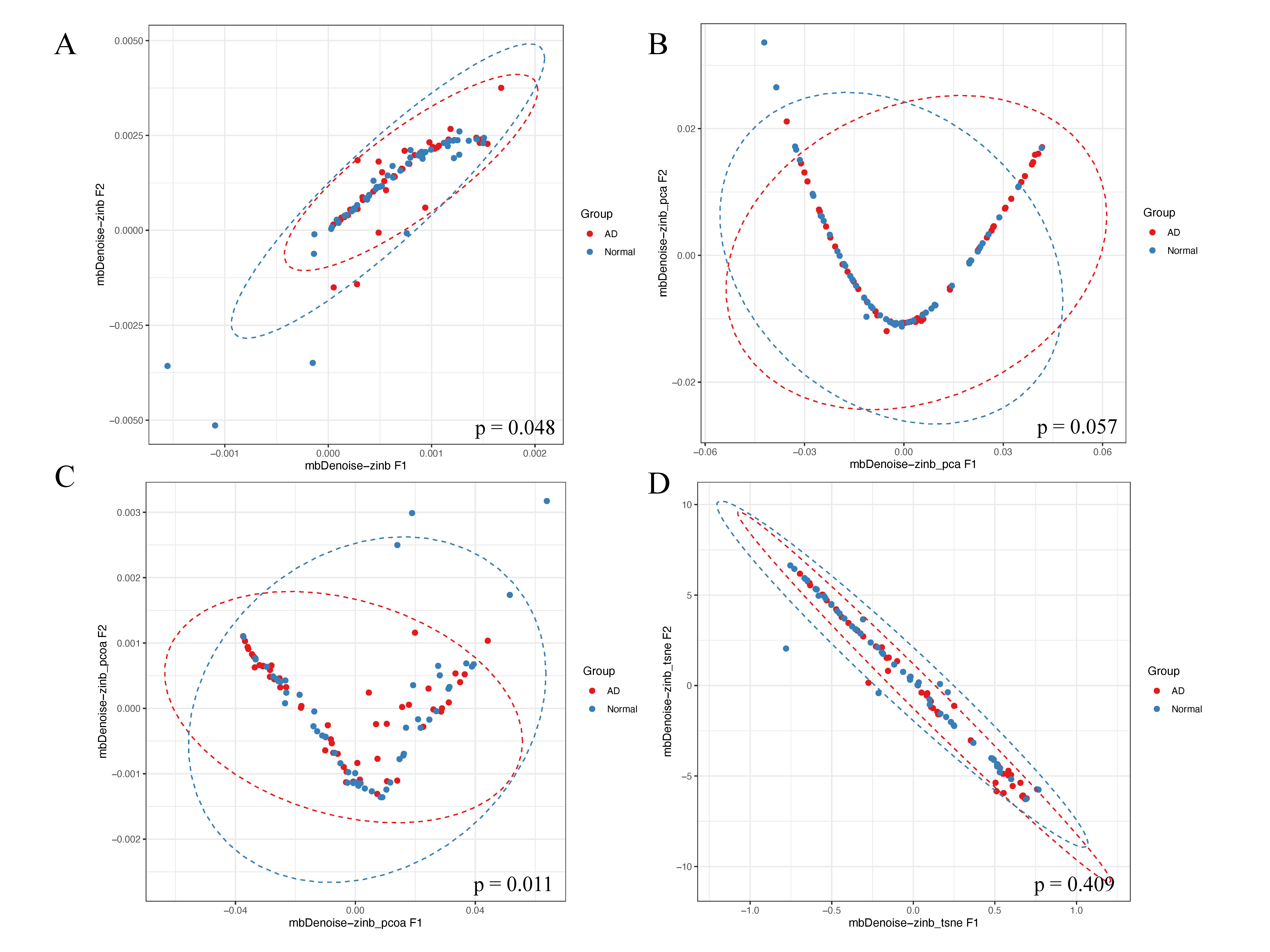

Supplement: Supplementary file 1 [file ijms-24-12856-s001.zip › FigureS2.jpg]

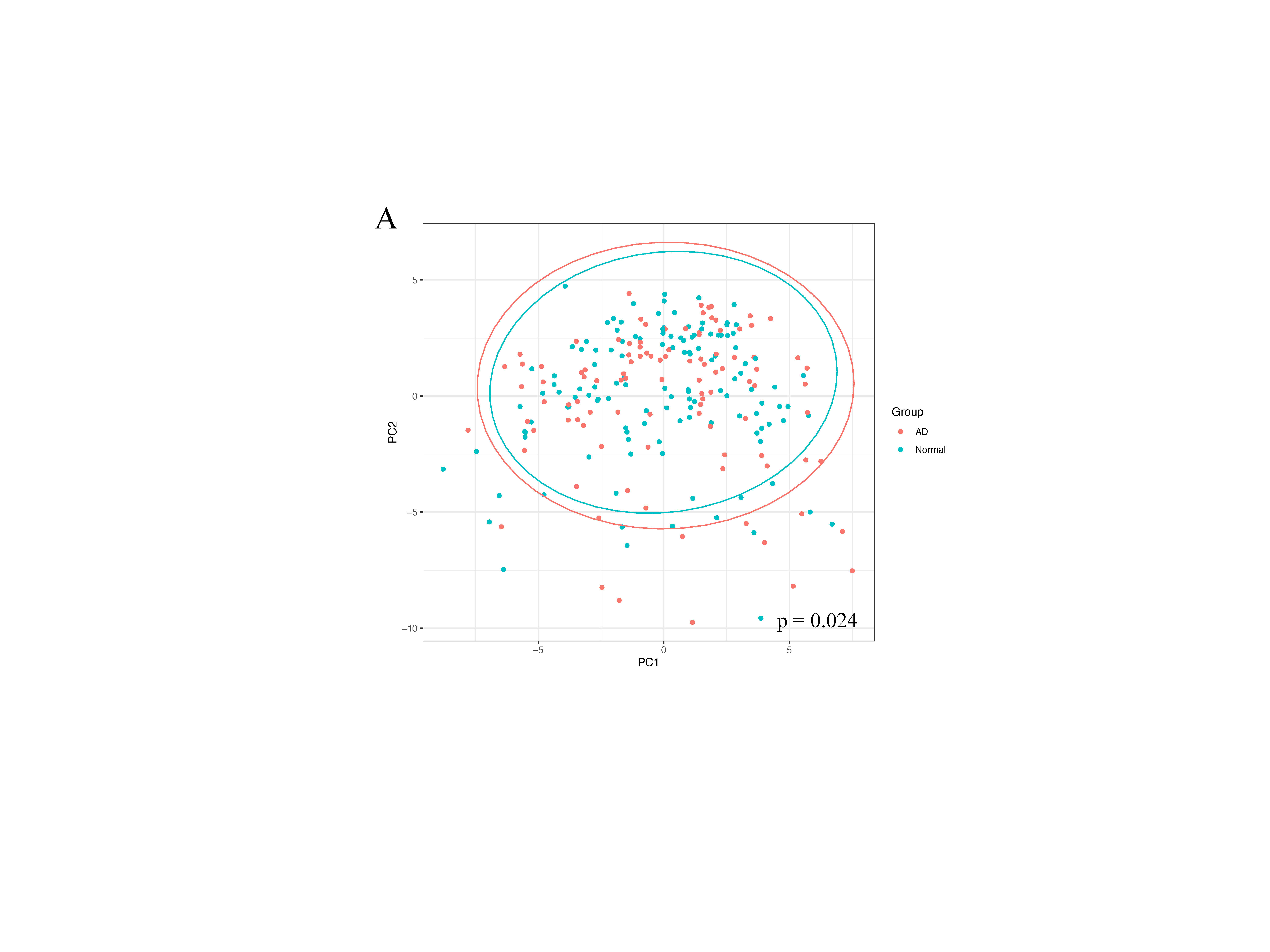

Supplement: Supplementary file 1 [file ijms-24-12856-s001.zip › FigureS3.jpg]

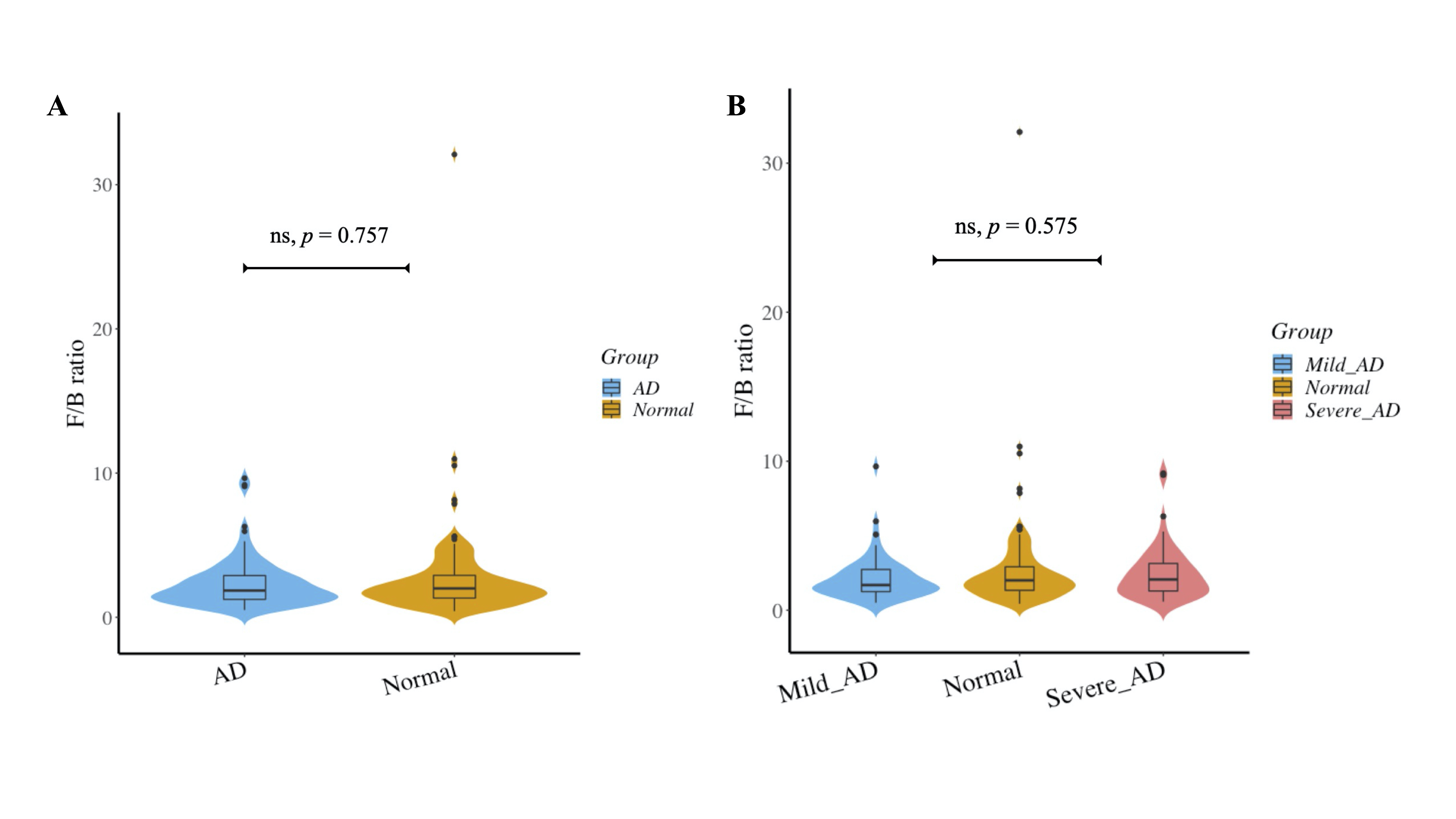

Supplement: Supplementary file 1 [file ijms-24-12856-s001.zip › FigureS4.jpg]

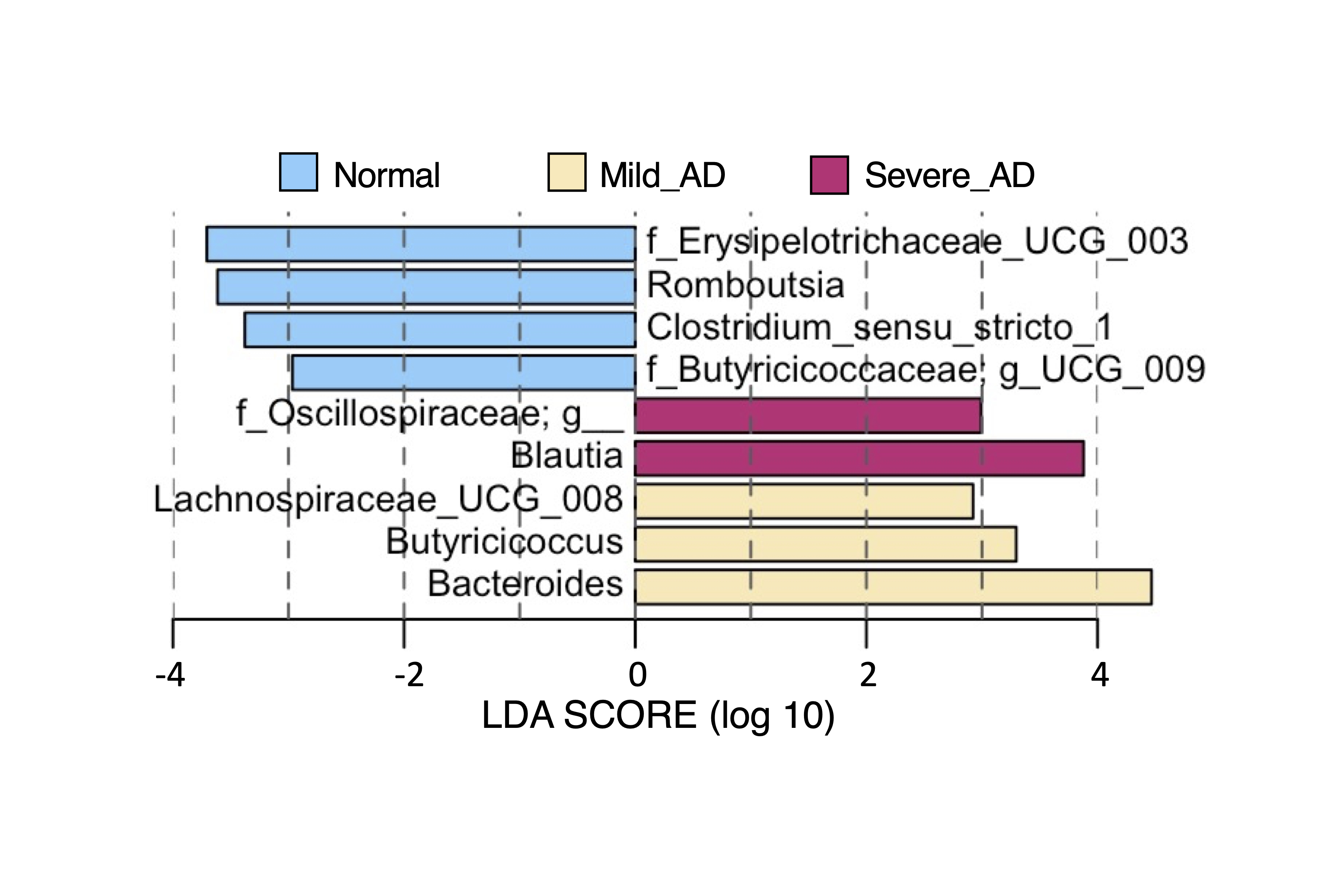

Supplement: Supplementary file 1 [file ijms-24-12856-s001.zip › FigureS5.jpg]

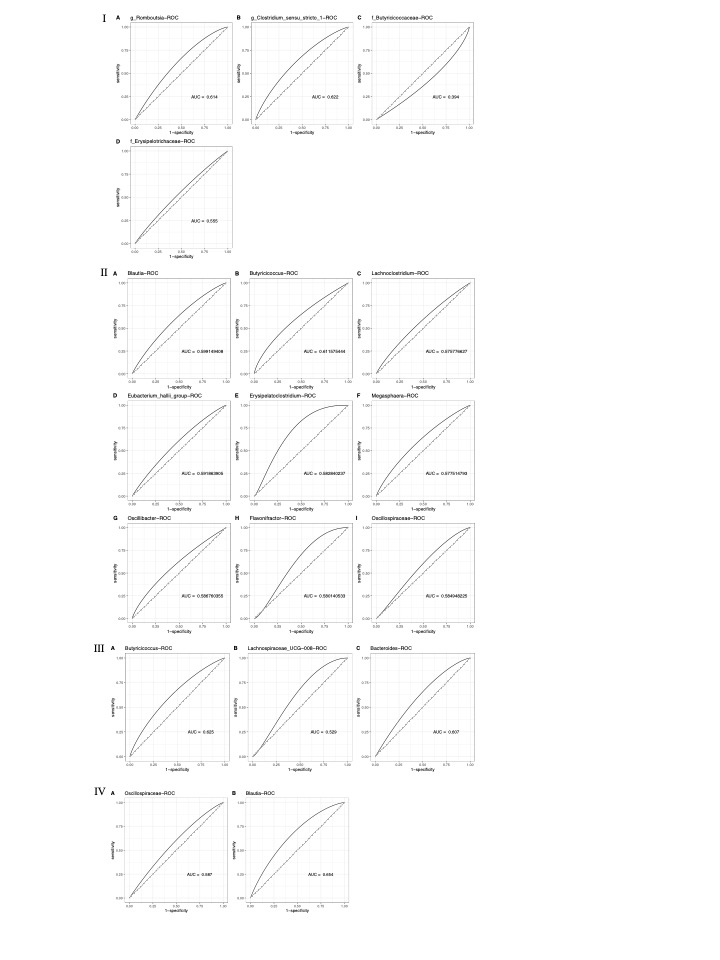

Supplement: Supplementary file 1 [file ijms-24-12856-s001.zip › FigureS6.jpg]
